# Projection of participant recruitment to primary care research: a qualitative study

**DOI:** 10.1186/s13063-015-1002-9

**Published:** 2015-10-20

**Authors:** David White, Daniel Hind

**Affiliations:** Clinical Trials Research Unit, University of Sheffield, Regent Court, 30 Regent Street, Sheffield, S1 4DA UK

**Keywords:** Qualitative research, Primary care, General practice, Participant recruitment, Recruitment projection, Planning, randomised controlled trials, Clinical trials

## Abstract

**Background:**

Recruitment to clinical trials remains a challenge, particularly in primary care settings. Initial projections of participant recruitment need to be as accurate as possible in order to avoid the financial, clinical and ethical costs of trial extensions or failures. However, estimation of recruitment rates is challenging and often poorly executed, if attempted at all. We used qualitative methods to explore the experiences and views of researchers on the planning of recruitment in this setting.

**Methods:**

Participants had registered accrual to a UK-based primary care research study between April 2009 and March 2012. We conducted nine interviews with chief investigators or study managers, using a semi-structured topic guide. Analysis was conducted using the framework approach.

**Results:**

Three themes are presented: 1) the factors affecting recruitment rates, 2) the use of planning techniques, and 3) influences on poor estimation. 1) A large number of factors affecting recruitment rates were discussed, including those relating to the study protocol, the clinical setting and the research setting. Use of targeted mail-outs to invite apparently eligible individuals to participate was preferred in order to eliminate some of the uncertainty in the recruitment rate associated with opportunistic clinician referrals. 2) The importance of pilot work was stressed. We identified significant uncertainty as to how best to schedule trial timelines to maximise efficiency. 3) Several potential sources of bias involved in the estimation of recruitment rates were explored and framed as technological, psychological or political factors.

**Conclusions:**

We found a large number of factors that interviewees felt impact recruitment rates to primary care research and highlighted the complexity of realistic estimation. Suitable early planning of the recruitment process is essential, and there may be potential to improve the projection of trial timelines by reducing biases involved in the process. Further research is needed to develop formal approaches that would be suitable for use in this setting.

**Electronic supplementary material:**

The online version of this article (doi:10.1186/s13063-015-1002-9) contains supplementary material, which is available to authorized users.

## Background

Recruitment to clinical trials remains a problem. A review of large UK-based multi-centre trials funded by the Health Technology Assessment (HTA) and Medical Research Council (MRC) reports that 45 % required an extension [1]. It is in the interests of trial funders that projections of recruitment provided by trial teams at the point of funding are as accurate as possible in order to avoid the financial, clinical and ethical costs of trial extensions or failures, yet little research has been done exploring estimation of the time required.

Recruitment projection can be challenging, if attempted at all. Researchers tend to use simple unconditional methods, where the time required is estimated by dividing the sample size by the projected monthly recruitment across all centres. More sophisticated models have been developed, but all are heavily dependent on the estimated parameters used [2] and will therefore, inevitably, be influenced by biases. When tasked with estimating the time required to complete a project, individuals tend to be overly optimistic even when they are aware that previous projects have overrun [3]. A manifestation of this cognitive bias in clinical research, sometimes known as ‘Lasagna’s Law’ [4, 5] or ‘Muench’s Third Law’ [6], is that researchers are often excessively optimistic about the number of people who are available and will need to be approached in order for a study to reach full accrual.

A clinical setting that faces particular recruitment challenges is primary care [7, 8]. A 2007 survey of 39 UK-based primary care trials found that fewer than one-third of the trials were recruiting to schedule [9]. Medical interventions have historically been tested in secondary care settings, and only relatively recently, have significant attempts been made to increase the quantity of research undertaken through primary care [10]. Many general practitioner (GP) practices remain either unable or unwilling to prioritise recruiting to research. Specific issues repeatedly identified in the literature include a lack of GP equipoise, resulting in a lack of willingness to randomise [11–15]; insufficient time to undertake the necessary tasks required to recruit participants [11–13]; and a lack of experience in research participation, resulting in unrealistic expectations [13, 16]. GP practices are small businesses and receive no core funding for research. Thus, where research activities have been inadequately costed, practice profits - and therefore partners’ incomes - are reduced, resulting in a disincentive to engage and prioritise the work [17].

Efforts have been made by organisations to aid recruitment in this setting, including work by the National Institute for Health Research (NIHR) Primary Care Research Network and Primary Care Research Recruitment Methods Group, who have published advice for researchers [18]. Such guidance is helpful, but in describing the large range of factors to consider when planning the recruitment process, this guidance highlights the complexity of estimating the time required when taking into account project-specific variables. Both the research design (including method of referral, intervention type and population) and the setting (including clinician engagement, experience and workload) will affect the speed at which recruitment takes place.

In this article, we report on qualitative research undertaken to explore the experiences and views of researchers on the projection of recruitment to clinical trials in primary care and consider how processes could be improved. We have two bases for ordering our findings. First, we use key concepts suggested by previous observations made in the medical and health science literature about how researchers project recruitment to trials. Second, inspired by the Reference Class Forecasting (RCF) model, we group explanations for the inaccuracy of recruitment projection under banner headings: ‘technical’, ‘psychological’, or ‘political-economic’ [19]. RCF is rooted in prospect theory [20], which states that:

- errors of judgment are frequently systematic and predictable rather than random, that is, they reflect bias, not confusion;
- experts make the same kinds of errors of judgement as laypeople;
- an awareness of one’s biases does not, by itself, enable a more accurate assessment of a situation.

RCF, ‘a method for de-biasing forecasts’, provides a conceptual basis for those who are aware of the problem to critically evaluate their circumstances and act on that evaluation.

## Methods

### Approach and rationale

Although recruitment is often studied quantitatively, for instance through investigating consent rates, when researchers wish to understand attitudes towards a phenomenon, the processes involved and the barriers to success, qualitative methods are more appropriate [21]. Our approach is phenomenological in that we aim to distil experiences of an issue [22, 23] - in this case the difficulties and opportunities associated with recruitment to trials by general practitioners. Our rationale is pragmatic [24]: we are less concerned with building or testing theory than the ‘conceivable practical consequences’ [25] of different lines of action, and providing a basis for ‘organising future observations and experiences’ [26]. In other words, we hope to guide future researchers and funders by describing the experiences of investigators who had already planned and undertaken studies in primary care.

Before commencing data collection, we searched the literature for papers from which we could build an initial thematic framework about recruitment projection specifically. Papers about recruitment strategies have been very common for over thirty years [27], but those that discuss recruitment projection are rare. We used free text terms such as ‘recruitment projection’ and ‘Lasagna's Law’ in a MEDLINE search, together with pearl growing and citation tracking to find papers. We used seven papers [28–34] to inform our initial topic guide (Additional file 1) and thematic framework. Table 1 shows the key themes of the framework as they relate to the literature identified.

**Table 1:** Key themes of the *a priori* framework, as they relate to the literature

*A priori* thematic framework	Agras I	Agras II	Hunninghake I	Hunninghake II	Ederer	Collins	Weintraub
Protocol factors							
Population and eligibility criteria	X	X		X	X	X	X
Recruitment methods used		X	X	X		X	
Complexity, burden, attractiveness of protocol				X			X
Setting factors							
Clinician equipoise				X			
Priority of research and workload	X			X		X	
Research culture and experience			X	X			
Variation between practices		X				X	
Changes in the clinical environment							X
Estimation							
Choice and quality of data used							X
Over-optimism					X	X	X
Planning							
Pilot work	X		X			X	
Feasibility work	X		X			X	
Projections	X	X	X	X	X	X	X
Contingency	X		X	X			

### Researcher characteristics and context

The research team consisted of the authors: DW and DH. DW is currently a trial manager and proposal development assistant at Sheffield Clinical Trials Research Unit (CTRU), who had previous experience in qualitative interviewing as part of a post-graduate degree, for which he conducted this research. DH is Assistant Director of Sheffield CTRU. We are both researchers with previous experience recruiting to clinical trials and are interested in the subject for professional reasons. As such, we had existing thoughts about the subject based on personal experience, prior to undertaking the study. All interviewees were working in the same field as the study team.

The interviewer, DW, had existing or previous professional relationships with four of the interviewees, who were more senior colleagues. The literature discusses advantages and disadvantages of conducting research with colleagues or peers [35]. The researcher may benefit from an understanding of the setting [36] and may bring experience that helps to interpret the findings and make them more meaningful [37]. We were also aware of the potential for such pre-existing relationships to affect the interviewer/interviewee dynamic, even though the subject matter under discussion was not particularly controversial, nor personal. We ensured that all questions in the topic guide were asked to all participants. We discuss other issues involved in interviewing colleagues or peers in the ‘ethical issues’ section of these methods.

### Sampling

A list of potentially relevant studies was obtained by searching the UK Clinical Research Network (UKCRN) portfolio database, with assistance from South Yorkshire Comprehensive Local Research Network (CLRN). Eligible participants worked on studies that were (a) UK based, (b) marked as General Practice or had received Primary Care Trust R&D approval, and (c) had registered accrual between April 2009 and March 2012. We took a pragmatic approach to sampling from this list, as we had limited time available to complete the study. Therefore, individuals on the list with links to the department of the study team or working in departments with links to the study team were initially prioritised. A list of 22 potential participants was then purposively sampled in order to reflect differences in intervention type, population, recruitment methods and, recruitment success. Fourteen investigators and study managers in total were invited via email to take part in an interview. Potential participants were provided with an information sheet and consent form with reply slip. Of those invited, 10 took part. One interview included two participants (both the chief investigator and study manager); thus, nine interviews were conducted in total.

With a relatively homogeneous participant group, nine interviews can be adequate to understand common perceptions and experiences, thereby achieving thematic saturation [38] (as distinct from other forms of saturation [39]). A formal assessment of whether saturation had occurred or of the stopping criteria for qualitative data collection was not employed [40]. After seven interviews we decided that insufficient data was available on trials that had tried to recruit incident populations, and for this reason, we recruited two additional participants who satisfied this criterion. While we felt that no new major themes were arising after nine interviews, the final decision to suspend recruitment was largely based on resource availability.

### Participant characteristics

Key characteristics of the study sample are presented in Table 2. We interviewed a roughly equal mix of investigators and study managers, and seven out of 10 were involved in the design of their study. A range of the characteristics purposively sampled for was achieved. Participants worked at institutions in four cities in the South-West, South-East and North of England. Target sample sizes of the studies under discussion ranged from 100 to 30,000. Most studies were funded by NIHR.

**Table 2:** Characteristics of the study sample

Interviewee characteristics				
Gender	Male	Female		
	6	4		
Study role	Investigator	Manager		
	6	4		
Involved in study design	Yes	No		
	7	3		
Study characteristics				
Study design	Randomised	Non-randomised		
	7	2		
Intervention	Therapeutic	Prevention	Screening	Other^a^
	5	2	1	1
Complex intervention	Yes	No		
	6	3		
Population	Acute	Chronic	Other^b^	
	2	5	2	
Recruitment method/s	Direct referral	Letter	Media	
	4	8	1	
Recruited to planned timescale	Yes	No		
	4	5		
Funder	NIHR	MRC	Charity	
	7	1	1	

^a^Other: medications adherence

^b^Other: healthy adults; high cardiovascular risk

### Ethical issues

Recruitment of colleagues or peers to research can present particular ethical challenges [35]. Potential participants may feel coerced into taking part [41] or may be concerned about the confidentiality of their participation amongst peers, and consequently, participants may alter their responses to questions. With this in mind, we provided all potential participants with a detailed participant information sheet and consent form. It was made clear that participation was voluntary, that no reason had to be given for declining participation and that the participant was free to withdraw at any stage without giving a reason. Participants were free to choose the location of the interview and consent was re-checked before it took place, including re-acquiring permission to digitally record the interview. Participants were informed that data would be fully anonymised. Given our intention for the reports of the research to be read by fellow members of the research community, we removed from published quotations all information that we considered could lead to the identification of the participant. Where data were removed, we were mindful to maintain the meaning behind accounts [42]. Pre-anonymised data were only seen by the interviewer, DW, who transcribed and anonymised the interviews.

Ethics approval for the research was granted by the NRES Committee Yorkshire and the Humber – Sheffield (10/H1308/12). All interviewees were university employees who were interviewed on university premises or by telephone.

### Data collection

Interviews were conducted by DW, either over the phone (*n* = 4) or face-to-face at the interviewees’ workplace (*n* = 5) and lasted between 28 and 65 min (median 40 min). Informed consent was obtained from all participants prior to participation. A paper consent form was either returned in the post prior to the scheduled telephone interview or completed with the participant in person. All interviews were audio-recorded and no-one else was present in the room. There were no repeat interviews. Interviews were semi-structured and informed by a topic guide (Additional file 1). Table 3 demonstrates how example questions from the topic guide relate to the main themes of the *a priori* framework. Interviewees were asked to discuss in depth their experiences on one study, but were also encouraged to draw on other experiences where relevant. Interviews were staggered, allowing later interviews to be informed by emerging themes. Transcripts were not returned to interviewees for comment, and interviewees did not provided feedback on findings.

**Table 3:** Example questions as they relate to the main themes of the *a priori* framework

	Example interview questions
Protocol factors	How did you approach potential eligible participants for recruitment into your study?
	Applying the eligibility criteria in practice, what did you find? Was it straightforward?
	Explore protocol regime and attractiveness to patient
Setting factors	It is often said that to be involved in a clinical trial, investigators and participants should be in equipoise, that is, genuinely uncertain about which treatment is better. Did you find that you had people involved who expressed preference for one treatment (patients) or conviction that one was better (clinicians)?
	Did any of these factors affect your own study: Staff availability (any particular times?) Practice busy? Different centres recruiting at different rates? Why do you think this is?
	Did anything in the clinical, organisational or policy environment change over the course of your study that affected recruitment?
Estimation	Some previous research has shown that when investigators are planning participant accrual to research, they are often overly optimistic in terms of the rate at which they expect this to happen. In your experience, generally, have you found this to be the case?
	Did you base your accrual estimates on: A clinical audit? Previous research? An estimate (whose?)? Was that realistic
	What information or knowledge did you not have when planning the trial that would have been most beneficial in projecting recruitment to the study? Would this have been available?
Planning	Was any pilot work undertaken, or a feasibility stage incorporated into the research? Were projections changed?
	Is there anything you can think of that would make it easier to project recruitment to studies generally?

### Data analysis

Analysis was conducted by DW, using the National Centre for Social Research Framework approach [43]. We used Framework analysis because it allows enough flexibility for analysts to identify subjects of known importance as coding categories from the outset and to combine them with other concepts that emerge from the data during inductive analysis. The five ‘key stages’ of framework were followed: familiarisation, identifying a thematic framework, indexing; charting, and, mapping/interpretation.

Data were transcribed verbatim, anonymised and imported into NVivo (QSR International v9.2). All data were stored electronically on a secure server. Familiarisation was achieved by reading and re-reading transcripts, considering participants’ accounts in light of the initial thematic framework that drove the design of the study, with notes being taken on new categories emerging from the data. For example, a theme of *a priori* interest was recruitment methods used; subthemes within this category were derived by reading the transcripts. A further review of the literature was also conducted to aid this process, with papers on specific primary care recruitment issues being utilised [11–16, 44–52]. For example, papers describing the use of database searching to aid recruitment [45] helped to give additional context to the data emerging on this subject. We also adopted the conceptual framework suggested by RCF to categorise areas of bias that can affect the planning of projects [19], which were defined as psychological, technical or political. Technical factors refer to the appropriateness, availability and quality of data sources; psychological factors refer to human biases; and political factors refer to the influence of funding mechanisms and strategic misrepresentation (deliberate underestimation).

**Fig. 1:**
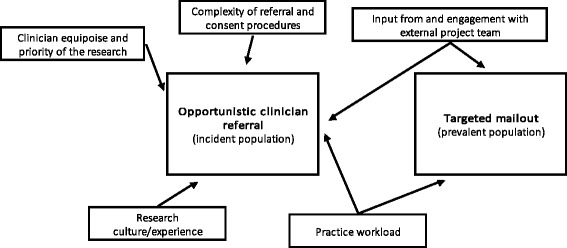
Factors identified as affecting accrual rates when using two alternative methods of recruitment

Following this process, a new framework was drawn up in NVivo. All data in the transcripts were then coded against the new framework, with continual refinement of categories occurring, some being merged where insufficient data were present. Coded data were then summarised using NVivo matrices, linked to the relevant quotations. Given the large number of categories, completed charts were printed out on paper to aid viewing and interpretation of the data, which were compared within and between participants. Where participants had very strong views on a subject or where there was considerable agreement or disagreement between participants was noted. Ideas emerging from the accounts were mapped out on paper to aid interpretation. For example, the relation between the recruitment method used and the influences on recruitment success was mapped out, as demonstrated in Fig. 1.

## Results

### The complexity of factors affecting recruitment rates

‘The smaller you break down a target, the smaller the parcels become, the more likely there is to be variation on it’. (H, Study Manager)

The interaction between the numerous factors influencing recruitment affects the ability to successfully project recruitment rates. Protocol factors were broadly thought to be within the control of the research team at the design stage, with setting factors largely outside of their control. The following are just some of the factors highlighted.

#### The protocol

Of particular concern for interviewees were that more complex eligibility criteria increase the difficulty in estimating numbers [4, 5, 28, 53].

‘What percentage of people have a severe mental illness at any one time, maybe eight or twelve percent, and another exclusion criteria might be a BMI over forty-five, so maybe five percent of people have that, but are they the same five, do you add those figures together or is there an overlap… you get an initial list of potentially eligible people and it gets slashed by fifty, sixty percent once you start putting exclusion criteria in’. (E, Investigator)

The comparative difficulty of recruiting an incident, as opposed to a prevalent, population, was highlighted by all the interviews. Where incident cases of acute conditions are required - and thus where opportunistic clinician referral is the only option - both recruitment, and also the ability to predict recruitment, become more difficult.

‘Incident cases are very different to looking for lists of people who have a particular condition already, because that’s a block of people that are just ready to approach, whereas… the incidence rate… can really slow you down… it’s, ‘oh yeah we get five or six of those a month’ and then when you actually do it in practice you only get one or two a month, so our study’s going to take three times as long as we thought’. (E, Investigator)

‘We couldn’t use any basis for calculations because it’s just really dependent on the rate at which you could get staff to make patients aware of this trial’. (G, Investigator)

Some interviewees thought that using multiple recruitment methods was ideal in order to increase the chances of success, although this may add an additional layer of uncertainty to estimates.

#### The clinical setting

The heavier the reliance on opportunistic clinician referrals, the greater will be the impact of the clinical setting on recruitment rates (Fig. 1). Factors stated as particularly important were clinician equipoise, practice workload, research experience, and input from and engagement with the external project team.

Recruitment rates achieved could be greatly affected by the willingness of the clinicians involved to recruit eligible patients, sometimes manifesting in failure to refer patients, or referral of an inappropriately selective group of people.

‘And what happened was that once they knew they were referring patients to [the intervention], they tended to choose slightly different patients because they had their own views about whether [the intervention] would be useful’. (A, Investigator)

In the following case, a list of eligible patients provided by the GP was smaller than expected: ‘And some may have just excluded patients on the basis of just, never seeing somebody perhaps. We went back and searched again in one case didn’t we, I seem to remember, because we didn’t believe the results.’ (C, Study Manager) Determining the reasons behind poor clinician referral rates is not necessarily straightforward. Clinicians often sound interested and do not deliver.

‘I’m deeply sceptical of healthcare professionals to directly refer people into trials and I’m always pleasantly surprised when I’m proved wrong in that respect, and I guess I was sort of proved right in this one, that healthcare professionals weren’t desperately keen to refer their participants to the trial… they sound interested when you talked about it, but then that very rarely converted into patient referrals’. (G, Investigator)

Overenthusiasm to recruit could also cause problems with ‘some bending the criteria and so you end up with patients that actually shouldn’t be in the study.’ (B, Study Manager) This could be put down to a lack of experience undertaking research. The following example illustrates the problem.

‘What we did identify was that the practices didn’t always apply the eligibility criteria,, a GP was supposed to go through the list of people who turned up on the searches and check them against the eligibility criteria and there were several cases where people were not excluded who should have been… so there, so what we’ve learned to do from that is to tighten up on the training of the people at the practices to say, ‘look, that’s really important, we don’t want to waste people’s time and get them in here and do all these blood tests and then find out that they weren’t eligible’. (E, Investigator)

Considerable variation between GP practices had been observed, with those centres that were more interested in and had more experience of research, both thought to recruit better and have more realistic expectations. Therefore, basing predictions on the performance of the more experienced practices (that the investigator is used to working with) could cause problems: ‘It was the case that the practices in [name of area] weren’t as research ready, as it were, as the ones in [name of area], so we possibly were a bit overoptimistic’ (D, Investigator). This study manager thought the safest approach was to stick to the research experienced practices: ‘I hesitate now going to a practice that hasn’t done anything before, because I don’t think they realise how much they have to do basically’ (I, Study Manager).

#### The research setting

The UKCRN facilitates both a topic-specific and a primary care research network and was introduced to provide an infrastructure to support recruitment and retention of research participants. There was agreement amongst interviewees that these networks have been helpful by improving access to GPs and patients. However, variations in procedure between networks and a lack of feedback and data on recruitment were seen as barriers to successful recruitment projections. ‘From the point of view of overall recruitment to similar types of studies [feedback and data on recruitment] would be really useful information’ (H, Study Manager). Several interviewees thought that research networks could improve the planning process, by providing basic data on key variables.

‘I think a good thing that could be done perhaps is to use the primary care research networks…to produce more information on their practices, their research practices, they could centrally run searches on them, across ten or twelve research practices as a kind of planning service, project planning and if they could keep just basic data on demographics and population size and age bands and things like that from their practices they could help a bit with that’. (E, Investigator)

Many UK research funders will only contribute to activities they consider to be directly associated with research, as defined by Department of Health Guidance Note HSG(97)32 and successor documents [54]. It is still a matter of surprise and frustration to many researchers that the definition of research activity excludes the identification of eligible patients - separately funded through ‘service support costs’ - and experimental treatments - again, separately funded through ‘excess treatment costs’ [55, 56]. Considerable delays are caused in the acquisition of these costs, as well as research and development approvals, which, in the UK are acquired from each participating institution, separately from, and subsequent to, Research Ethics Committee approvals [57–61]. Geographical variation in access to support/treatment costs and in R&D approval times made estimation of study timelines problematic. ‘It’s now seven months and we still haven’t got [R&D approval], so god knows when we’ll get it, it’s just miserable.’ (D, Investigator) Where insufficient time was built into the grant, delays could affect recruitment, especially in trials involving seasonal conditions. ‘If the R&D approval had come through in a timely fashion, they could have started a year earlier’. (H, Study Manager).

### Use of planning techniques

‘I think you need to be as realistic as possible, I don’t think you’re ever going to get an accurate picture. You do your projected, predicted recruitment rates and you can try and stick to that, but you’ve got to be flexible’. (I, Study Manager)

The difficulties involved in planning a clinical study were highlighted in the interviews. Various methods were explored and sometimes differing opinions expressed in relation to the utility of such methods in estimating study timelines.

#### Pilot and feasibility work

There was broad consensus that pilot work was of benefit in projecting recruitment to a full trial. ‘You could almost justify doing a ‘pilot pilot’ study or something just to look at recruitment’ (E, Investigator). One interviewee had encountered an unexpected problem, which took 6 months to resolve and which, had it occurred during a full trial, would have had more severe consequences. ‘If we hadn’t done the pilot we would have been really screwed’ (D, Investigator). Not all interviewees had conducted pilot work, and some regretted this. However, some did raise caution that recruitment rates observed in pilot studies may not be representative of those in a full trial. ‘[The pilot] all happened very simply, but they were practices we’d worked with before’ (F, Investigator).

Those with particularly complex trial protocols or interventions and whose research involved a lot of buy-in from either patient or GP practice regretted not consulting more with those that would be involved by undertaking early qualitative work to determine feasibility and interest.

‘It was a complex trial and we had lots of components to it… I’d go back and do a little more research with people and say ‘what do you think of this design’…. they’re very helpful and you know they’ll soon say if something’s not going to work. You can’t predict from that how well it will work, but it does stop the disasters happening’. (C, Investigator and Study Manager)

Those that highlighted the importance of such early feasibility work did so more within the context of improving the trial design and intervention generally, rather than using qualitative work (as distinct from pilot work) to assist in estimating trial timelines or recruitment projections. While the importance of qualitative work was highlighted, those interviewees with more straightforward trial designs and standard interventions had considerably less to say on the subject.

#### Projections, scheduling and contingency

The majority of interviewees agreed that formulating recruitment projections during the planning stage was a useful process (see also, ‘influences on poor estimation’). Those against projections focused on the futility of getting the estimate right:

‘I don’t know how things would have been made different if you haven’t really done x, y and z by when you said you were supposed to… if I’d have made Gantt charts or anything, well I just don’t see the point because I would have missed them all the time [laughs]’. (H, Study Manager)

The frequently employed counter-argument was that, ‘it’s worth doing, because if you don’t do it then you don’t know you’re not on target’ (I, Study Manager). There was a general acceptance that timelines would be likely to change, that it’s important to be flexible, and that recruitment is rarely achieved at a steady rate.

‘You usually have a recruitment curve, so you plot out what you’re expecting to do over the time period, and it’s always a straight line which I find quite surprising because usually it’s not a straight line, you start off slowly and then you build up and then it peaks and then drops off a bit towards the end, ’cause yeah you learn what the barriers are as you go along and often it doesn’t go the way you expected and you have to take measures to correct and get back onto the curve

*And so with this study did you, what sort of a curve did you have for your recruitment then?*

Um, well just a straight line [laughs]’. (E, Investigator)

Events outside the control of the study team, for example a change in the diagnosis or management in the population being studied, can affect an otherwise sensible schedule. The success of the NHS Quality and Outcomes Framework (QOF), introduced to reward and incentivise GPs in the management of chronic disease, was thought to have reduced the pool of poorly managed patients eligible for this study of a chronic condition: ‘We thought [the eligibility criteria] were broad to start off with…we’d assumed on the basis of previous work there would be quite a large pool of people to recruit’ (C, Investigator). Researchers believed that building in contingency to the trial timelines was sensible, but that the implications for costs often made this impractical.

### Influences on poor estimation

‘I think the way it normally works is they have their target number and their timescale and they work backward’. (I, Study Manager)

Technical, psychological and political factors were all identified in the interviews. In many instances, it is hard to extricate technical and political influences from psychological ones - as biases are inherent in any human decision making - so there was some overlap between the themes.

#### Technical

Interviewees had used a variety of data sources to inform estimates, all with strengths and limitations. The most commonly used data source was recruitment data from previous research, usually from the interviewee’s own or colleagues’ research. This is the most easily accessible data but has weaknesses.

‘We'll say to the collaborators and including ourselves, 'has anyone done a study with a similar population before in primary care and what kind of recruitment rates do you get' and try and base it on facts as much as possible. But even then you get two different studies and one will have a recruitment rate three or four times the other one and you say ‘OK which estimate shall we use’. (E, Investigator)

The temptation here is to go with the more optimistic figure, as discussed in the next section, ‘psychological influences’.

To determine patient numbers, data derived from clinician estimates were considered to be the least reliable, whereas looking at practice data directly was preferable. Talking to GPs ‘you get big variability and you don’t really know from the outset if you can trust that information or not, whereas with the database you’re bypassing all of that’ (F, Investigator). Interviewees thought practice data were reliable and accurate for items collected for the QOF, but that other data could be much less so. In addition, availability and variability between practice systems can be a barrier. However, such data can at most answer one piece of the jigsaw - the number of potentially eligible patients - and cannot help with other variables affecting recruitment rates.

Ultimately this interviewee thought the technical process of projecting recruitment was not that hard, but that access to the right data was the main barrier: ‘I don’t think it’s a difficult thing to do, to estimate recruitment rates, but it relies on you having relevant data and that’s the difficulty’. (E, Investigator)

#### Psychological

All interviewees thought that investigators are generally overly optimistic when estimating recruitment but that the reasons varied.

‘If you’re a GP, for example, if you have an interest in back pain… people with back pain may come to see you preferentially. So… you think ‘oh well I’m seeing two or three a week for this condition and I’ll times that by five [for the other GP partners], oh that’s fifteen a week and OK even if we recruit half of those we’re going to get seven in a week’ but what he or she doesn’t know is that the partners are only seeing perhaps one a week because all of the other people are coming to you’. (D, Investigator)

Most interviewees had at some point used recruitment figures from previous research to inform estimates for a current project. However, when using such data, there is a tendency to inappropriately ‘anchor’ [62] estimates by focusing on positive past experiences and failing to consider important differences between the studies.

‘The [previous study]… per patient it was definitely better funded than this one… it was hideously optimistic to expect this sort of study to be done on such a small budget’. (H, Study Manager)

Although all interviewees thought investigators were generally overly optimistic, several explicitly stated that on their own studies they try to be as pessimistic as possible (B, E and G). Interviewees often framed their own experience in positive terms, sometimes stating that initial targets had been met when it later transpired this was not strictly the case. Such statements could be interpreted as examples of recall bias or the ‘Lake Wobegon Effect’, a tendency for individuals to overestimate their achievements relative to the average [63].

#### Political

Several researchers acknowledged tending towards optimism in the projections provided to funding bodies, due to institutional pressures to secure the funding.

‘You want to put together a grant application which is realistic as well in terms of being able to win that grant and so you can’t have things going on for years, so it’s hard to balance it properly so you’re not going back asking for extensions’. (C, Investigator)

‘When you plan the project you have an eye to the funding, funders want value for money… you could say ‘oh, well we’ll recruit one patient a month and take a hundred months and we’ll have a full time researcher employed all that time’, but that’s not really going to work. There is a certain amount of pressure to fit the funding that’s available and compress that into a timescale’. (E, Investigator)

Occasionally there was evidence that researchers were not intrinsically motivated to take the planning process seriously.

‘I think a lot of this planning and stuff is done more for the benefit of outside bodies, rather than actual running of the study… most of the time when you’re writing things like Gantt charts a lot of it is bullshit and you’re just doing it because you’ve got to do it’. (H, Study Manager)

## Discussion

### Principal findings

This qualitative study has explored three key themes relating to the projection of recruitment to clinical trials in primary care settings. In agreement with previous work [8, 9, 44], a large number of factors affecting recruitment were identified. This highlights that realistic estimation of recruitment rates is complex. Where prevalent cases are required, use of practice databases and targeted mail-outs is considered preferable in order to maximise recruitment success and aid estimation of the number of eligible patients for the study [8, 45]. By avoiding the requirement for individual GPs to refer patients to the trial, targeted mail-outs bypass many of the problems of the clinical setting highlighted in these findings and previous research [11–16] - specifically; lack of GP equipoise, conflicting time priorities and insufficient research experience. Where opportunistic GP referrals are required, the variability in recruitment performance between and within GP practices makes estimation of recruitment challenging, and consequently, some of our respondents voiced a preference for exclusively using experienced practices. Interventions aimed at GPs to increase awareness of general research principals and to explain the position of equipoise driving the trial design could be of benefit to planning recruitment rates, as this might reduce some of the uncertainty around individual GP performance. One of our interviewees had randomised practices to receive additional information about the trial treatments, and reported that those who’d received this referred twice as many patients. Use of such interventions could be explored further.

We identified major issues relating to the research setting, which were manifested in delays to securing NHS costs and obtaining approvals to undertake the research. The severity of delays experienced makes the projection of timelines particularly difficult and, in many cases, impossible to anticipate in advance. Planned changes to the approvals process, via the recently established Health Research Authority, will bypass the requirement for individual local organisations to give R&D approval and may enable more reliable estimates of the time required.

Early planning of the recruitment period has long been identified as an important step in clinical trial implementation [64]. Pilot and feasibility work were both thought to be important aspects of the planning stage, for different reasons - pilot work specifically to aid in projecting recruitment to a full trial and feasibility work to improve complex trial processes and interventions. Our interviewees had conducted little in the way of early feasibility work and there was little recognition of this as an aid to projecting recruitment specifically. The use of pilot data for projections, while helpful, should be treated with caution due to variations between rates achieved on a small scale versus when rolled out in a full trial. With regards to the use of specific planning techniques, we identified some ambivalence as to the benefits of formulating recruitment projections, although most interviewees thought this an important exercise. There was also uncertainty as to how to best schedule trial timelines to maximise efficiency, while including contingency for unanticipated problems, such as issues in the research setting described above.

Exploration of the possible influences on poor estimation of trial timelines, framed within the explanations used by Flyvbjerg [19], resulted in some interesting findings, not previously explored qualitatively in the clinical trials literature. Technical issues around poor data quality and availability, as well as inappropriate use of data, were identified. The finding that data obtained from GP estimates was considered the least reliable was perhaps unsurprising, given that estimates provided by individuals may be more prone to bias, and given other findings concerning the barriers created by a lack of GP experience in research. In addition, we found that investigators might not always use the most effective sources of data, prioritising previous research conducted within their team, over wider distributional data. Whether such a preference is due to bias or insufficient availability of appropriate data is not always clear. Kahneman and Tversky [3] found that people have a tendency to be ‘insufficiently sensitive to distributional data, even when such data are available’ (ch. 2, p. 2). Examples of psychological bias were clearly present in the data, often resulting in overly optimistic projections. In addition, some evidence of political bias was discovered, with pressures to secure funding potentially impacting on recruitment estimates. Some interviewees were explicitly aware of a balance between securing funding and providing realistic projections to funding bodies.

### Strengths and limitations of the study

This is the first study to our knowledge that has investigated specifically the projection of recruitment to clinical trials in primary care. Other studies have focused on methods to improve recruitment to trials in this setting, but we believe there has been little recent research exploring the methods used to undertake the planning stages of trial recruitment. Use of qualitative methods has enabled a detailed exploration of the experiences and attitudes of those involved in the process.

We interviewed a relatively small sample of researchers, and there were some limitations in the sample inclusion criteria. Only Chief Investigators and Study Managers were interviewed - the inclusion of Clinical Trials Unit proposal development staff may have resulted in a broader range of views - and the study was limited to discussion of non-commercial research. Prioritising invitations to people with links to the department of the study team is a limitation, though we included a range of studies with varying designs and populations and interviewed staff at several institutions across the United Kingdom.

It is possible the interviewer gave a degree of reverence to interviewees, who were largely more senior personnel working in the same field, all of whom had direct or indirect links to the study team. There are potential advantages and disadvantages to interviewing peers, as discussed in our methods. Perhaps the most sensitive area of discussion, the political motives behind projections, was not an *a priori* question in the interview schedule, but was volunteered by a number of interviewees, indicating a confidence in discussing such issues openly in the interview environment.

### Implications for researchers

In line with previous literature, we recommend that researchers should plan early for recruitment [4, 28], ensuring sufficient consideration is given to the methods used to recruit. Teams should closely monitor progress against pre-set thresholds and act early where changes to the project plan are required.

Improving the estimation of project timescales would result in better use of public funds and may also reduce existing cynicism among some researchers as to the benefit of formulating projections. Researchers may be best placed using a variety of data sources where possible and favouring distributional data over personal experience. Researchers could consider use of methods similar to RCF as an aid to reducing unconscious biases involved in the process. Of the three forms of bias explored in this paper, technical and psychological biases could be reduced by using such an approach [19]. To aid this, it will be important to improve the availability and accessibility of detailed recruitment data from previous trials. Combined with current attempts to ensure trial registration and publication of trial protocols [65–67], this would enable researchers to more easily access relevant distributional data pertinent to their planned trial. Improving the general culture of research within primary care and strengthening the relationship between practitioner and researcher could enable an easier path to recruitment projection. Keeping patient records with research in mind and provision of easier access to such data could improve the information used to inform future studies. As suggested by one of our interviewees, the research networks could assist in this process.

Where deliberate political biases exist, independent challenge and accountability may help to counter these. Recent guidance [68] stresses the importance of this when allocating public funds, suggesting that overly optimistic projections are caused in part by a failure of governance. In agreement with van der Wouden and colleagues [8], our results suggest that funding bodies should use a consistent and systematic approach in requesting evidence to back up estimates provided by trial teams. When doing so, they should consider whether the applicants have used appropriate methods for the task. For example, where prevalent populations are required, have the applicants provided data derived from practice databases to back up the estimated number of eligible patients? Have they provided recruitment data from a number of previous studies investigating similar populations and interventions?

### Implications for future research

This exploratory research was limited to recruitment in the primary care setting. Additional qualitative research could be undertaken in this and other clinical settings, further exploring the utility of using formal methods to aid projection of recruitment. Systematic approaches similar to RCF could be developed, such as that employed by Cooper and colleagues for diabetes prevention and therapy trials [69]. Their approach involved calculating pooled recruitment rates, with 95 % confidence intervals, for those screened, eligible and randomised as a function of those approached. Similar approaches have also been taken in systematic reviews of recruitment to dementia studies [70] and behavioural trials [71]. Further research is needed to develop a consensus-based formal approach.

Research could also be undertaken to determine whether systematic techniques could be applied to the estimation of participant attrition. Fabricatore and colleagues systematically reviewed attrition rates in pharmacological weight loss trials [72], whereas Villeneuve and colleagues reviewed drop-out rates from psychosocial treatments for schizophrenia [73]. Anecdotally, these studies have contributed to planning further research in their own field, and systematic reviews of data points, such as recruitment and attrition rates, might also assist in the planning of future trials in primary care.

## Conclusions

A large number of complex factors can affect recruitment rates to research in the primary care setting, especially where opportunistic clinician referrals of patients with acute conditions are required. Suitable early planning of the recruitment process is essential, and there may be potential to improve the projection of trial timelines by reducing biases involved in the process. Further research is needed to determine how formal processes could be developed for this purpose.

## Additional file

Additional file 1:
**Interview topic guide.** Full interview topic guide used in the interviews. (DOC 34 kb)

## Abbreviations

GP: general practitioner
MRC: Medical research council
NHS: National Health Service
NIHR: National Institute for Health Research
QOF: quality and outcomes framework
RCF: reference class forecasting
R&D: research and development
UKCRN: United Kingdom Clinical Research Network
