# Supplementary material for: Projection of participant recruitment to primary care research: a qualitative study
Source: Trials. 2015 Oct 20;16:473. doi: 10.1186/s13063-015-1002-9 (PMC4615323; doi:10.1186/s13063-015-1002-9)
Supplement: Additional file 1: — Interview topic guide. Full interview topic guide used in the interviews. (DOC 34 kb) [file 13063_2015_1002_MOESM1_ESM.doc]

1. Could you describe your study for me:

*Prompt:* Number of years of funding/funder

Start and end date of study/recruitment

Study design

Population

Interventions

Outcomes

Required Sample size

2. Did you attain your required sample size?

3. Did you require an extension to the scheduled period over which you were recruiting? And if so why did you need an extension? Was it primarily to do with recruitment?

*Prompt:* Did you require additional funding as a result?

4. Some previous research has shown that when investigators are planning participant accrual to research, they are often overly-optimistic in terms of the rate at which they expect this to happen. In your experience, generally, have you found this to be the case?

With the benefit of hindsight, in this particular project, do you think you would have made different assumptions as to how many participants you would recruit… per month for example?

*Prompt:* Did you base your accrual estimates on:

A clinical audit?

Previous research?

An estimate (whose?)?

Was that realistic?

How easy was data to come by?

*Prompt:* Was any pilot work undertaken, or a feasibility stage incorporated into the research?

Were projections changed as a result?

5. How did you approach potential eligible participants for recruitment onto your study?

*Prompt:* Did you use more than 1 method?

How successful were these?

Were there any problems with specific methods?

6. Did you record the numbers of non recruited patients at any time during your study?

*Prompt:* Were there more than you expected?

7. Applying the eligibility criteria in practice, what did you find?

*Prompt:* Was it straight forward?

In hindsight do you think the criteria eligibility criteria were overly restrictive?

*Prompt:* Often eligibility criteria exclude people from a study who in practice would be eligible for the treatments.

*(explore)* Protocol regime – unattractive to the patient?

8. It’s often said that to be involved in a clinical trial investigators and participants should be in equipoise, that is genuinely uncertain about which treatment is better. Did you find that you had people involved who expressed preference for one treatment (patients) or conviction that one was better (clinicians)?

*Prompt* If so, did this/or in what way do you think this influenced recruitment?’

9. Previous research has found that investigators often find their recruitment rate estimates to be problematic because of:

- Selective memory and wishful thinking
- GP records not sufficient
- Patients moving, retiring, dying, recovering
- Disease classification changing over time

Did you encounter any of these problems?

10. There are some other factors we have identified that might affect patient recruitment. Did any of these factors affect your own study?

*Prompt:* Staff availability (any particular times?)

Practice busy

Different centres recruiting at different rates? Why do you think this is?

11. Did anything in the clinical, organisational or policy environment change over the course of your study which affected recruitment?

*Prompt:* Clinical guidelines

Practical issues within the practice

Staff

Prevalence of the clinical condition

Competing priorities (prompt: industry-funded studies with financial incentives to GPs for recruitment versus NHS R&D sponsored work; local interests overriding the implementation of your study; government targets such as the QOF).

12. Do you feel there was enough time in the development of the study to sufficiently plan for recruitment and formulate an accurate projection?

*Prompt:* if not why not

13. What information or knowledge did you not have when planning the trial that would have been most beneficial in projecting recruitment to the study?

*Prompt:* would this have been available?

Is there anything you can think of that would make it easier to project recruitment to studies generally?

14. With your experience /hindsight what do you think would be the main piece of advice in terms of planning for recruitment that you would give to trial teams when designing clinical trials and putting together grant applications?

15. Is there anything else you want to add?
